# Examining the relationship between autistic spectrum disorder characteristics and structural brain differences seen in anorexia nervosa

**DOI:** 10.1002/erv.2910

**Published:** 2022-05-15

**Authors:** Daniel Halls, Jenni Leppanen, Jess Kerr‐Gaffney, Mima Simic, Dasha Nicholls, William Mandy, Steven Williams, Kate Tchanturia

**Affiliations:** ^1^ King's College London (KCL), Institute of Psychiatry Psychology and Neuroscience (IoPPN) Psychological Medicine London UK; ^2^ King's College London Centre for Neuroimaging Sciences London UK; ^3^ South London and Maudsley NHS Foundation Trust London UK; ^4^ Division of Psychiatry Imperial College London London UK; ^5^ Division of Psychology and Language Sciences University College London London UK; ^6^ Psychology Department Illia State University Tbilisi Georgia

**Keywords:** anorexia nervosa, autism spectrum disorder, gyrification, structural MRI, surfaced‐based morphometry

## Abstract

Cortical differences have been reported in Anorexia Nervosa (AN) compared with healthy controls (HC); however, it is unclear if Autism Spectrum Disorder (ASD) characteristics are related to these cortical differences. The aim of this study was to examine if structural measures were correlated to ASD traits in AN. In total 184 female participants participated in the study; 57 acutely underweight AN participants (AAN), 59 weight‐restored participants (WR) and 68 HC. Participants underwent structural magnetic resonance imaging as well as completing the Autism Diagnostic Observation schedule, second edition to examine ASD characteristics. Group differences in curvature, gyrification, surface area, thickness, global grey matter and white matter were measured. Correlation and regression analysis were conducted to examine the relationship between cortical measures and ASD characteristics. Two decreased gyrification clusters in the right post central and supramarginal gyrus and decreased global grey matter were observed in the AAN group compared to HC and WR. No correlations between ASD traits and structural measures existed. Our results suggest structural differences seen in individuals with AN do not appear to be related to ASD characteristics.

## Highlights

- Though widespread structural differences have been reported in anorexia nervosa the relationship to autistic traits is under‐researched and unclear.
- In this study autistic characteristics were not correlated to structural measures or structural differences in anorexia nervosa.
- Possible reasons for our results including variation in underlying biological process between anorexia nervosa and autism, as well as heterogeneity within anorexia nervosa are discussed, along with strategies for future work.

**Abbreviations**
AAN: Acutely Underweight Anorexia Nervosa
ADOS‐2: Autism Diagnostic Observation Schedule, 2^nd^ edition
AN: Anorexia Nervosa
ASD: Autistic Spectrum Disorder
BMI: Body Mass Index
CC: Cortical Curvature
CSA: Cortical Surface Area; CT, Cortical Thickness
EDE‐Q: Eating Disorders Examination—Questionnaire
FEW: Family‐Wise Error
GLM: general linear model
HC: Healthy Controls
LGI: Local Gyrification Index
SRB: Stereotype and Repetitive Behaviours
Tukey‐HSD: Tukey honest significant differences
WR: Weight Restored

## 1 INTRODUCTION

Anorexia Nervosa (AN) is a life‐threatening eating disorder, characterised by low body mass index (BMI), an intense fear of weight gain and undue influence placed on body shape and weight (APA, 2013). Incidences of AN are rising (Keski‐Rahkonen & Mustelin, 2016) and despite AN having a significant mortality rate (Arcelus et al., 2011), the neurobiology of the disorder is still poorly understood (Kaye et al., 2013). However, accumulating evidence has demonstrated that structural brain differences exist in individuals with AN (Bernardoni et al., 2018; King et al., 2018; Leppanen et al., 2019). Numerous studies have reported that underweight individuals with AN have decreased grey and white matter volumes compared to healthy controls (HC, Fonville et al., 2013; Seitz et al., 2015; Nickel et al., 2018). Cross‐sectional studies and a recent meta‐analysis suggest that no global volume differences exist between weight‐restored individuals with AN and HC (Lázaro et al., 2013; Bang et al., 2016; Nickel et al., 2018; Seitz et al., 2018).

Structural imaging studies have also demonstrated group differences in local cortical surface measures in underweight individuals with AN compared to HC (Bernardoni et al., 2016; Leppanen et al., 2019). Local cortical surface measures are considered to be more informative of structural architecture than local volume‐based analysis, as surface‐based measures model genetically independent properties of cortical architecture, such as volume and folding (Winkler et al., 2010). Cortical volume consists of two independent cortical surface‐based measures, cortical thickness (CT), representing the number of neurons in neuronal columns and cortical surface area (CSA), representing the number of neuronal columns (Panizzon et al., 2009; Winkler et al., 2010). Cortical folding, which is important due to its relationship to connectivity (Mota & Herculano‐Houzel, 2012), consists of cortical curvature (CC) which represents the sharpness of the sulci and the gyri and the local gyrifcation index (LGI), which compares the area of visible and buried cortex within the sulci (Luders et al., 2006; Schaer et al., 2008). All four of these measures CT, CSA, CC and LGI, have been demonstrated to be altered in individuals with AN. Widespread reduction in LGI and widespread increase in CC have been demonstrated in underweight individuals with AN compared with HC, which normalises with weight restoration (Bernardoni et al., 2016, 2018). This suggests an inverse relationship between CC and LGI, such that as gyri ‘deflate’ they become sharper (as reflected by increased CC) resulting in less cortex buried within the sulci (represented by decreased LGI, Bernardoni et al., 2018; Leppanen et al., 2019). Widespread reduction in CT has been consistently reported in underweight individuals with AN compared to HC (Favaro et al., 2015; Bernardoni et al., 2016; Miles et al., 2018) which resolve with weight–restoration (Bernardoni et al., 2016). Localised reduction in CSA in the temporal and frontal cortices has been reported in underweight individuals with AN (Leppanen et al., 2019). However, this finding has been disputed (Miles et al., 2018) and the effect of weight‐restoration on CSA has not been explored.

Despite some consistency of these structural findings, it is unclear what clinical features have a relationship with these biological changes. Eating disorder symptoms have a limited relationship to LGI (Bernardoni et al., 2018) and have no correlation to CT recovery (Bernardoni et al., 2016), as well as exhibiting no relationship to CSA (Leppanen et al., 2019) or to volume (Fonville et al., 2013). Depressive symptoms have been shown not to be correlated to the normalisation of CT (Bernardoni et al., 2016) and grey matter volume changes are independent of both obsessive compulsive and depressive symptoms (Fonville et al., 2013). The only measure that has consistently been shown to be related to structural differences is BMI. Seitz et al. (2015) and Lavagnino et al. (2016) found that BMI was correlated to grey matter volume and CT respectively. While Bernardoni et al. (2016, 2018) found increase in BMI resulted in a normalisation of LGI and CC.

There has however, been a limited number of studies examining the relationship between autistic spectrum disorder (ASD) characteristics and structural changes seen in individuals with AN. Behavioural evidence suggests that ASD characteristics are over represented in individuals with AN (Kerr‐Gaffney et al., 2021; Westwood & Tchanturia, 2017), with individuals with AN scoring similar to women with ASD in social difficulties, restricted interests and repetitive behaviour (Kerr‐Gaffney et al., 2021). Structural imagining studies in autistic individuals have show similar alterations to individuals with AN, such as decreased global volume (Ha et al., 2015), decreased regional CT (Pagnozzi et al., 2018) and decreased LGI (Schaer et al., 2013) reported. This could suggest a possible trans‐diagnostic process between AN and ASD. The importance of exploring the link between ASD and AN is highlighted in a recent systematic review which showed that adaption of eating disorder treatment for autistic people improves treatment outcomes (Li et al., 2021). The limited evidence examining the relationship between ASD characteristics and structural differences suggests that AN participants compared to HC have reduced grey matter volume in the right temporal sulcus, which was correlated to ASD characteristics (Björnsdotter et al., 2018). As far as we are aware, no study has examined the relationship between surface‐based measures and ASD characteristics in people with AN.

Therefore the aim of this study was to explore if group differences in CT, CSA, LGI, CC and global volumes were correlated to ASD traits in women with AN. To address whether ASD traits are associated with starvation or represent a trait seen also in weight recovery, a sample of young, acutely underweight AN (AAN) participants, HC and weight‐restored participants (WR) was used. To examine if a possible potential relationship between structure and ASD traits existed, correlations between global volumes and group‐based differences to ASD traits were explored. Our hypothesis was that group differences in structural measures would be correlated to ASD traits.

## 2 METHODS

### 2.1 Participants

191 female participants were recruited, which were stratified into AAN (57 participants), WR (60 participants) and HC (73 participants). All participants underwent screening using the structured clinical interview for DSM 5 researcher version for any major psychiatric co‐morbidities (First et al., 2015), and checked for eligibility. Participants were eligible if they were right‐handed, aged 12–27, had no history of serious brain trauma, learning disabilities or neurological impairment, and no MRI incompatibility (pregnancy, claustrophobia and metal in or around the body which was unable to be removed). All participants gave written, informed, consent and the study was approved by National Research Ethics Committee (17/LO/2071). All research activities were conducted in accordance with the Declaration of Helsinki (2013).

Before the MRI scan, participants' height and weight were measured to calculate BMI (kg/cm^2^) to allow for group stratification. Individuals under 18 years old had their BMI adjusted for age, using percentage of median BMI for age and gender, with a cut‐off of 85% of median BMI being used for group stratification based on clinical guidelines (Royal College of Psychiatrists, 2012). AAN was defined as having a concurrent diagnosis of AN as defined by DSM‐5 criteria, with a percentage of median BMI of less than 85% for participants aged under 18 or a BMI of less than 18.5 for participants aged over 18. WR was defined as having previously been diagnosed with AN, but having had a BMI within the healthy weight range (18.5–25) if over 18 years old, or a percentage BMI of greater than 85% if aged under 18 years old at the time of enrolment and MRI scan. All patients with AN were recruited from the South London and Maudsley specialist Eating Disorders Service, South West London and St George's Eating Disorders Service, as well as Beat, the largest UK charity for eating disorders. This study was conducted independently from any clinical care participants were receiving and the authors did not have access to any patient records. HCs were defined as having no history of an eating disorder and not being underweight (BMI <18.5) for age at the time of the study. This resulted in three HCs being excluded due to being underweight for age, leaving 70 HC. HC participants were recruited from the local community, as well as King's College London staff and students.

### 2.2 Image acquisition

Images were acquired on a 3T GE MRI scanner, located at the Centre for Neuroimaging Sciences at King's College London. A high resolution T1 weighted image was taken, with the following parameters: echo time 3.016 s, repetition time 7.312 s, 1.2 mm slice thickness, field of view 270 mm, flip angle 11° and a matrix of 256 × 256 pixels.

### 2.3 Clinical and self‐reported measures

Before the MRI session, participants completed the Eating Disorders Examination‐ Questionnaire (EDE‐Q, Fairburn & Beglin, 2008). Participants also completed a demographic questionnaire, which included age and duration of illness and the National Adult Reading Test to measure IQ (Nelson & Wilson, 1991). Before the MRI scan, participants underwent the Autism Diagnostic Observation Schedule, second edition (ADOS‐2) to assess for ASD characteristics by a trained ADOS‐2 administrator. The communication and social, creativity, stereotyped and repetitive domains of the ADOS‐2 were used as a measures of ASD characteristics within our population.

### 2.4 Statistical analysis

#### 2.4.1 Clinical and self‐reported measures analysis

Group differences on the ADOS‐2 communication and social, creativity, stereotyped and repetitive domains, age, BMI, EDE‐Q global domain and IQ were examined with a one‐way ANOVA, or if the data did not meet the assumptions of one‐way ANOVA, a non‐parametric Kruskal‐Wallis test was used. When significance was found, Tukey honest significant differences (Tukey‐HSD) or if the assumptions where not met for Tukey‐HSD, Mann‐Whitney U tests corrected for multiple comparisons, were conducted to test for direction of significance. To test if illness duration differed between the AAN and WR groups a *T*‐test was conducted. All data was analysed in python 3.9.7.

#### 2.4.2 Neuroimaging data

Neuroimaging data was analysed using Freesurfer version 6.0 (https://surfer.nmr.mgh.harvard.edu/). Pre‐processing was done using the *recon ‐all* pipeline (using the *recon‐all –all* command) which has been extensively used in structural MRI analysis (King et al., 2018; Bernardoni et al., 2018; Leppanen et al., 2019; Cascino et al., 2020). This pipeline pre‐process the T1 images by motion correcting, intensity normalising, skull stripping, volumetrically labelling, segmentating and parcelling the images (see https://surfer.nmr.mgh.harvard.edu/fswiki/recon‐all for full details on the *recon‐all* pipeline). Once the images were pre‐processed, quality control of pre‐processed output was conducted by first visually inspecting the output for errors. All the images exhibited pial and white matter surface errors, which were corrected first manually by the authors using *recon* editing mode and then re‐ran through the *recon‐all* pipeline. The pre‐processed output was further examined for quality control issues using the ENIGMA Cortical Quality Control Protocol 2.0 (ENIGMA Cortical Quality Control Protocol 2.0, 2017). Cortical thickness and surface area were extracted from each participant for each of Freesurfer's areas of interest and examined for any outliers using R and Matlab scripts. Internal and external segmentation of the pre‐processed output was visually inspected and checked against ENIGMA quality control guidelines for potential errors. Finally histograms of group cortical thickness and group surface area were inspected to examine if group thickness and surface area followed a normal distribution. After quality control, two scans from the HC group, one from the WR and one from the AAN group had to be removed due to image acquisition errors, global segmentation issues and pathology, leaving 57 AAN participants, 59 WR participants and 68 HC (184 in total). This initial pre‐processing provided measures of thickness, curvature and surface area for each participant.

The next stage was to calculate the local gyrification index (LGI) using the rec*on‐all ‐localGI* command. Measures where then spatially smoothed, with thickness, surface area and curvature smoothed with a gaussian full‐width half maximum kernel set to 10 mm (using *qcache* command). For the LGI output, as the LGI is already relatively smooth (Schaer et al., 2012), a smoothing kernel of 5 mm (rather than 10 mm used for the other measures) was chosen to prevent excessive over‐smoothing of the LGI surface.

Following pre‐processing, calculation of the LGI and smoothing, the measures were assembled for group surface‐based thickness, area, curvature and LGI differences, by re‐sampling the spatially‐smoothed images into a single common space file (using the *mri_preproc* command). The general linear model (GLM) was then fitted to the each of the measures (using *mri_glmfit)* to examine for group based cortical differences. Age was initially added to the model however, caused severe levels of collinearity (see supplementary material), which has been shown to reduce the power of the analysis and make parameter estimates unreliable (Mumford et al., 2015). The reason for this collinearity is most likely the reflection of age in the group make up, as age‐adjusted BMI was used as a criteria for group selection. Therefore to correct this issue, age was removed from the model and parameter estimates were correlated to age, to examine the impact of age on structural measures. To correct for multiple comparisons, cluster‐wise correction was employed with Monte‐Carlo permutation simulation with 10,000 iterations (using the *mri_glmfit‐sim* command). An uncorrected cluster‐defining threshold of *p* < 0.0005 and a cluster‐wise corrected *p*‐value of <0.05 were used, in line with previous research (Leppanen et al., 2019). To account for Freesurfer analysing each hemisphere independently, the *p*‐value was also corrected using Bonferroni correction. When significance was found, direction of significance was ascertained using the Tukey‐HSD method from the extracted parameter estimates (due to the data being normally distributed). Parameter estimates from each group were also used to ascertain if the measure was increased or decreased as well as calculating effect sizes using Cohen's D.

To test for global volume differences, global volumes were extracted from the images (using the *asegstats2table* command). The volumes of interest were the global grey matter (TotalGrayVol on asegstats table) and white matter (the sum of cerebellum white matter and cerebral white matter on the asegstats table) volumes. Mean global thickness, LGI, CSA and CC were also calculated and extracted (using the *aparcstats2table* command). Group differences on global and cortical measures was examined using one‐way ANOVAs, except when assumptions for the one‐way ANOVAs where not met, then non‐parametric Kruskal Wallis tests were conducted. Tukey‐HSD tests were conducted to test for direction of significance except when assumptions for the Tukey‐HSD were not met, then post hoc Mann‐Whitney U tests were conducted with correction for multiple comparisons done by controlling the family‐wise error (FWE) rate using the Holm‐Sidak method (with *p*(FWE) < 0.05 considered significant).

To fully examine the effect of ASD characteristics on structural measures, a number of strategies were used. First, extracted cluster means from significant between‐group clusters were correlated using Spearman's test of correlation to the ADOS‐2 communication and social, creativity, stereotyped and repetitive domains, age, BMI and EDE‐Q global domain within each group. Illness duration was added for the AAN and WR groups. Correction for multiple comparisons was conducted using FWE, correcting for the number of tests across all groups (calculated using the Holm‐Sidak method with *p*(FWE) < 0.05 considered significant). Secondly, global measures, which included global grey and white matter volumes as well as mean LGI, CT, CSA and CC were correlated to the ADOS‐2 communication and social, creativity, stereotyped and repetitive domains as well as to age, BMI and EDE‐Q global domain within each group. Again illness duration was added for the AAN and WR groups and correction for multiple comparisons (corrected for the number of tests across all groups and all measures) was done using FWE with *p*(FWE) < 0.05 considered significant. Correlations analysis and parameter estimate exploration was done in python 3.9.7. Finally linear regressions were conducted with the ADOS‐2 communication and social, creativity, stereotyped and repetitive domains being the independent variables and CT, LGI, CSA and CC being the dependent variables. This was conducted within the AAN group using Freesurfer's *qdec,* which examines localised vertex disruption of surface measures. Correction for multiple comparisons was again done using permutation simulation. All analysis scripts can be found on github at https://github.com/WMDA/ASD_structure.

## 3 RESULTS

### 3.1 Self‐reported and clinical measures

Participants' clinical and self‐reported data is presented in Table 1. All groups differed on BMI, with the AAN group having the lowest, then the WR group and finally HCs. Both the WR and AAN groups had statistically significant higher EDE‐Q scores compared to the HC group but did not differ from each other. The AAN group had statistically significant higher ADOS‐2 creativity domain and ADOS‐2 communication and social domain scores compared with the HC group. The WR group had a statistically significant higher score on the ADOS‐2 stereotyped and repetitive domain, compared to the HC and AAN groups. A group difference for age was detected, however ad hoc Tukey‐HSD did not detect the direction of significance. No group differences were seen for illness duration or IQ.

**TABLE 1:** Participants self‐reported and clinical measures with Kruskal‐Wallis test for group differences and multiple comparisons corrected Mann‐WhitneyU test to test for direction of significance

	AAN mean (std)	WR mean (std)	HC mean (std)	Group difference	Direction of significance
BMI	16.38 (1.39)	20.32 (2.35)	22.89 (3.36)	*H* = 114.75	HC > AAN (*p* = < 0.01, *d* = 2.58)
				*p *= < 0.01	WR > AAN (*p* = <0.01, *d* = 2.13)
				eta = 0.67	HC > WR (*p* = <0.01, *d* = 0.98)
				*df* = 168	
Age (Years)	19.40 (2.83)	18.25 (3.51)	19.41 (3.37)	*H* = 6.72	–
				*p* = 0.04	
				eta = 0.03	
				*df* = 181	
EDE‐Q	3.35 (1.54)	2.87 (1.63)	0.59 (0.83)	*H* = 81.40	AAN > HC (*p* = < 0.01, *d* = 2.19)
				*p* = <0.01	WR > HC (*p* = < 0.01, *d* = 1.73)
				eta = 0.48	
				*df* = 167	
IQ	112.67 (7.64)	110.68 (7.64)	108.96 (6.94)	*H* = 0.5.02	–
				eta = 0.02	
				*df* = 166	
				*p* = 0.08	
ADOS‐2 communication and social domain.	3.66 (2.65)	2.91 (2.89)	1.55 (2.10)	*H* = 17.82	AAN > HC (*p* = <0.01, *d* = 0.99)
				*p* = <0.01	
				eta = 0.14	
				*df* = 111	
ADOS‐2 creativity domain.	0.77 (1.09)	0.48 (0.51)	0.27 (0.61)	*H* = 10.01	AAN > HC (*p* = <0.01, *d* = 0.56)
				*p* = <0.01	
				eta = 0.07	
				*df* = 111	
ADOS‐2 stereotyped and repetitive domain.	1.54 (1.27)	2.24 (1.36)	0.97 (1.01)	*F* = 10.53	WR > AAN (*p* = 0.04, *d* = 0.56)
				*p* = < 0.01	WR > HC (*p* = <0.01, *d* = 0.56)
				eta = 0.13	
				df = 111	
Illness duration (years)	3.69 (2.81)	3.74 (2.73)	–	–	–

Abbreviations: AAN, acute anorexia nervosa; ADOS‐2, autism diagnostic observation scale; AQ10, autism spectrum quotient; BMI, body mass index; df, degrees of freedom; EDE‐Q, eating disorders examination; HC, healthy controls; WR, weight‐restored anorexia nervosa.

### 3.2 Group differences in structural measures

#### 3.2.1 Global measures

**FIGURE 1:**
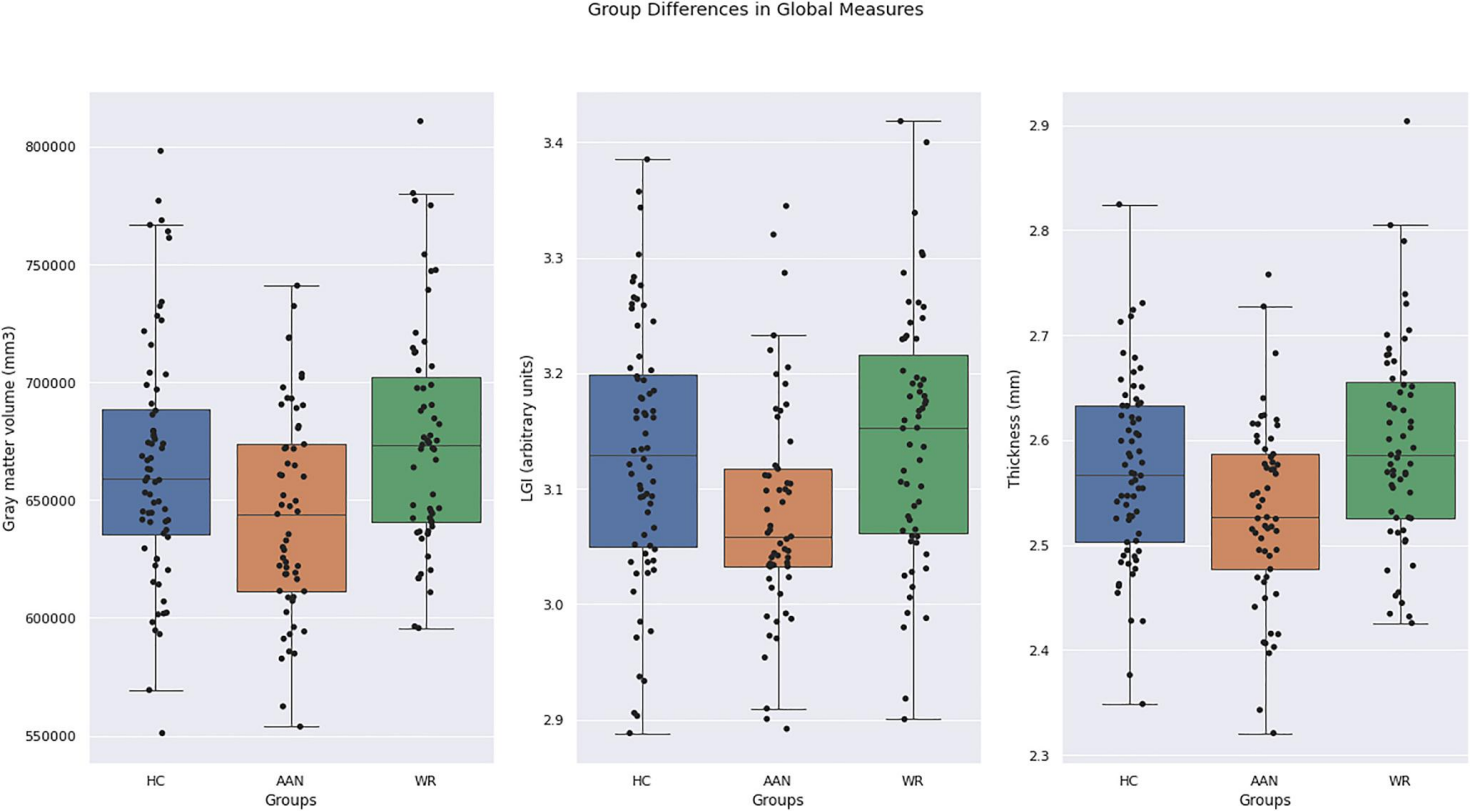
Plot of group differences in global grey matter, local gyrification index and thickness by group

A significant group‐based difference was detected for global grey matter (*H* = 11.27, *p* < 0.01, eta = 0.05, *df* = 181). This was between the AAN group and the WR and HC groups with the AAN having reduced global grey matter volume (mean = 644,339.00 mm^3^, std = 43,517.48 mm^3^) compared with WR (mean = 676,096.75 mm^3^, std = 48,021.51 mm^3^) and HC (mean = 665,213.69 mm^3^, std = 50,980.11) groups (see Figure 1), with a large and medium effect size respectively (*d* = 0.94, *d* = 0.58). No significant group differences were detected for cerebral white matter volumes (*F* = 0.57, *p* = 0.57, eta < −0.01, *df* = 181).

A significant group‐difference was shown for global LGI (*F* = 6.07, *p* = <0.01, eta = 0.06, *df* = 181) and global CT (*F* = 7.27, *p* = <0.01, eta = 0.07, *df* = 181). The AAN group had reduced LGI (mean = 3.08, std = 0.10) compared to the WR (mean = 3.14, std = 0.11) and HC (mean = 3.13, std = 0.11) groups with a small effect size for both (*d* = 0.28, *d* = 0.22 respectively). The AAN group had decreased CT (mean = 2.53, std = 0.09) compared to the WR (mean = 2.60, std = 0.10) with a small effect size (*d* = 0.29). No group differences were exhibited for CSA (*F* = 1.47, *p* = 0.23, eta = 0.02, *df* = 181) and CC (*F* = 2.08, *p* = 0.13, eta = 0.02, *df* = 181) (Table 2).

#### 3.2.2 Surface‐based measures

**FIGURE 2:**
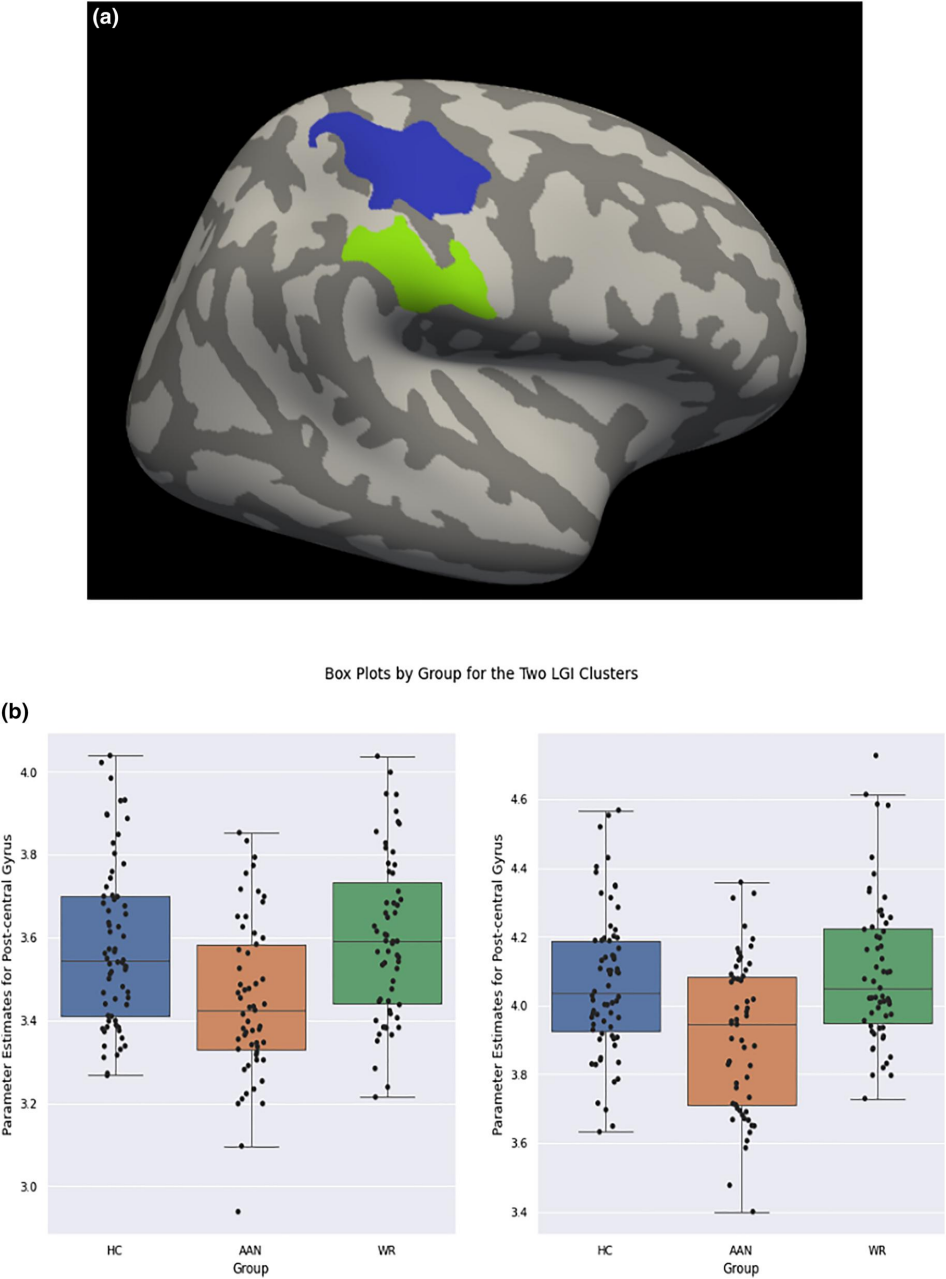
(a) GLM Group Level between all three groups for Local Gyrification Index. (b) Plot of parameter estimates from GLM by group

The GLM demonstrated a significant between‐group difference for the LGI in two right hemisphere clusters, located at the post‐central and supramarginal gyrus as shown in Table 3 and Figure 2. Post hoc Tukey‐HSD tests showed the AAN group had decreased LGI compared to WR and HC groups for both clusters with a medium effect size for both clusters (see Table 3 and Figure 2).

**TABLE 2:** Global Measures for each group

Measure	AAN mean (std)	WR mean (std)	HC mean (std)
Grey matter (mm^3^)	644,339.00 (43,517.48)	676,096.75 (48,021.51)	665,213.69 (50,980.11)
White matter (mm^3^)	446,351.16 (49,469.46)	449,085.34 (40,261.30)	454,645.40 (43,570.36)
Global LGI (arbitrary units)	3.08 (0.1)	3.14 (0.11)	3.13 (0.11)
Global cortical thickness (mm)	2.53 (0.09)	2.60 (0.10)	2.57 (0.09)
Global cortical curvature (arbitrary units)	0.13 (<0.01)	0.13 (<0.01)	0.13 (<0.01)
Global cortical surface area (mm^2^)	82,180.50 (6184.79)	84,101.20 (6455.55)	83,847.96 (6947.62)

Abbreviations: AAN, acute anorexia nervosa group; HC, healthy control group; LGI, local gyrification index; std, standard deviation; WR, weight restored anorexia nervosa group.

No other significant clusters were found for the LGI and no significant group differences were detected for CT, CSA, CC.

**TABLE 3:** Local gyrification index differences when controlling for age

Peak region	Cluster Max	Size (mm^2^)	Number of vertexes	MNI Co‐ordinates	*p* value (CWP)	Mean parameter estimates (std)	Direction of significance (Cohen's D)
Right post central	4.50	1864.78	4275	*X* = 36.1	<0.01	AAN: 3.45 (0.19)	HC > AAN (0.39)
				*Y* = −30.4		WR: 3.60 (0.20)	WR > AAN (0.44)
				*Z* = 61.3		HC: 3.58 (0.20)	
Right supramarginal	5.40	952.31	2347	*X* = 54.4	0.01	AAN: 3.91 (0.22)	HC > AAN (0.42)
				*Y* = −24.3		WR: 4.10 (0.22)	WR > AAN (0.52)
				*Z* = 33.9		HC: 4.06 (0.21)	

Abbreviations: AAN, acute anorexia nervosa; CWP, clusterwise *p* value; HC, healthy control; LGI, local gyrification index; MNI, montreal neurological institute; std, standard deviation; WR, weight‐restored anorexia nervosa participants.

### 3.3 Correlation analysis of structure and ASD characteristics

**FIGURE 3:**
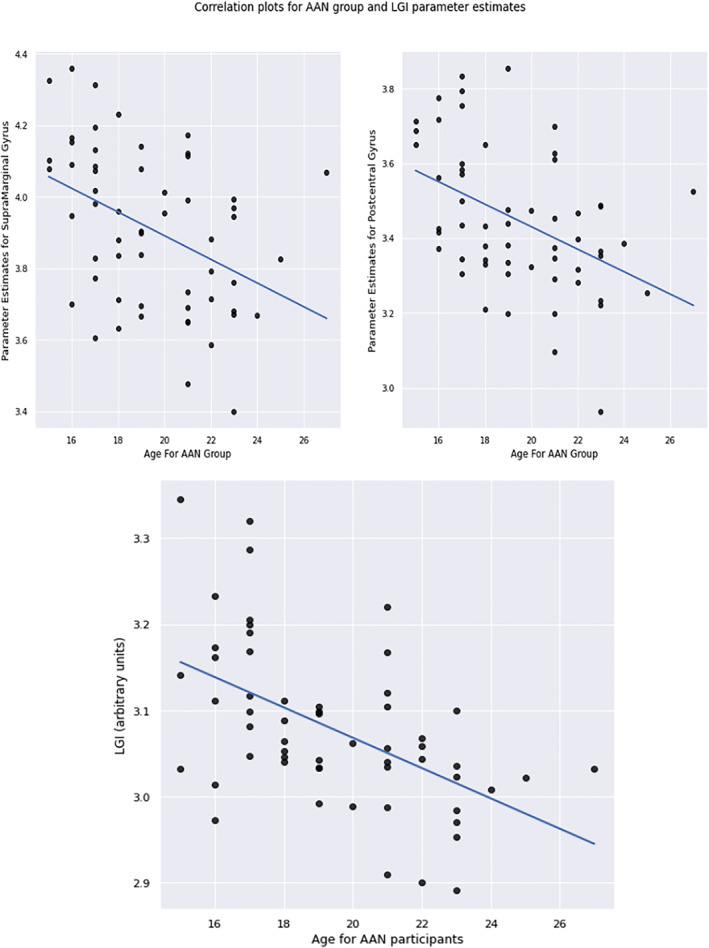
Correlation plot of Age to Local Gyrification Index within the acutely underweight group. (a) For the significant between‐groups clusters. (b) For the global volume

Extracted parameter estimates from the significant between‐groups LGI clusters showed a significant negative correlation for both clusters to age in the AAN group (*p* = 0.02, rho = −0.45 for the post‐central gyrus and *p* = 0.01, rho = −0.46 for the supramarginal gyrus, see Figure 3). No other correlations for any other measures or for any of the other groups were found.

In the AAN group, global LGI exhibited a negative correlation to age (*p* = <0.01, rho = −0.45, see Figure 3). No other measures were correlated to LGI for any of the groups and no correlations were shown for grey and white matter volumes, global CT, CC and CSA. The linear regressions with domains of the ADOS‐2 as the independent variables and CT, CSA, LGI and CC as the dependent variables in the AAN group demonstrated no significant results.

## 4 DISCUSSION

The aim of the study was to examine the association between ASD characteristics and structural differences seen in individuals with AN. Our main hypothesis was that ASD measures would be correlated to structural measures and differences seen in AN. Our data did not support this hypothesis.

Our behavioural results found that the AAN group had increased scores for ADOS‐2 domain scores in communication and creativity domains with medium to large effect size compared to HC group, with the WR group in‐between. This replicates previous work which has found that individuals with AAN have increased ADOS‐2 scores, similar to that of autistic individuals when compared to HC, with WR individuals lying between groups (Kerr‐Gaffney et al., 2021). Interestingly, our results demonstrated that WR individuals had increased stereotype and repetitive behaviours (SRB) compared to AAN and HC individuals with a medium effect size. Previous behavioural studies have been inconclusive of the role of SRBs in a possible AN and ASD link (Kerr‐Gaffney et al., 2020, 2018, 2016). One study directly comparing women with AN and autistic women, showed no difference between acutely underweight, recovered and autistic individuals on the ADOS‐2 stereotype and repetitive domain (Kerr‐Gaffney et al., 2021). However, results from graph theory analysis demonstrated little linkage between repetitive behaviours and eating disorder symptoms (Kerr‐Gaffney et al., 2020). A potential reason for this could be the use of self‐report questionnaires verses objective interviews. Our study and studies that have used the ADOS‐2 have found increased SRB scores (Kerr‐Gaffney et al., 2021) compared to studies using self‐reported measures (Kerr‐Gaffney et al., 2021). As autistic women and men have different repetitive interests (Lai et al., 2015), it may be that current tools are not sensitive enough to pick up differences.

Our structural imaging results did replicate previous reductions in global grey matter volume in underweight individuals and localised para‐central reductions in LGI seen in underweight participants in previous studies (Cascino et al., 2020). However, despite the AAN and WR group showing increased scores on all domains of the ADOS‐2, group structural differences were unrelated to ASD traits. Our results also suggest that the biggest driver of localised changes in LGI is age and that global measures are independent of eating disorder symptoms and illness duration. This fits in with previous research, showing that eating disorder symptoms and illness duration are unrelated to structural changes (Bernardoni et al., 2016, 2018).

A possible reason for a lack of any correlations to ASD traits is that in AN, ASD traits may manifest themselves through functional rather than structural differences. Recent work has shown that in acutely underweight individuals with AN, while undertaking a theory of mind task, domains of the ADOS‐2 were associated with an atypical blood oxygen level dependent response in the left dorsal posterior cingulate and right extra‐striate cortex (Leslie et al., 2020). However, counter to this explanation is work by Björnsdotter and colleagues who found that autistic traits were correlated with decreased volume in the left tempo‐parietal junction (Björnsdotter et al., 2018), suggesting that ASD traits in the underweight stage are related to structure.

Another possible explanation for the lack of correlations within the AN groups is the heterogeneity of AN. Recent work using cluster analysis has identified a number of different cognitive profiles in individuals with AN, including small potential ‘ASD like’ clusters (Kerr‐Gaffney et al., 2021; Renwick et al., 2014). It could be possible that within our AN sample a small number of individuals were in this ‘ASD like’ cluster, however this group was too small and under‐powered to find any observable effects in the correlation analysis. In addition to this, a correlation may be present between structural measures and ASD traits however, the effect was small it could not survive the correction for multiple comparisons. Correcting for multiple comparisons is known to reduce statistical power (Lee & Lee, 2018) and this may have resulted in false negative findings. Though this study tried to reduce the impact on power by using a step down Sidak approach to maintain statistical power (Lee & Lee, 2018), false negative findings cannot be ruled out.

Another possible reason why no correlations were seen between structural measures in AN individuals and ASD traits is the variation in the underlying biological processes of topological differences. Structural changes in AN seem to reflect nutritional status (Bernardoni et al., 2016, 2018, 2016; King et al., 2015) with some authors calling the global reduction a pseudo‐atrophy, rather than apoptotic‐induced atrophy (King et al., 2015). More importantly these topological differences shown in AN have been demonstrated as reversible (Bernardoni et al., 2016, 2018; Nickel et al., 2018; Seitz et al., 2018) which our results seem to support. Though autistic individuals may have similar structural differences to individuals in AN (Ha et al., 2015; Pagnozzi et al., 2018), a diverse range of molecular processes underpin these changes (Won et al., 2013). Structural changes viewed in autistic individuals are also not reversible, but follow a progressive path (Ha et al., 2015). Therefore, although the topological features seen in AN and ASD might superficially resemble each other, the underlying causes of these changes are different and that may explain why no correlations to structure and ASD traits were seen in this study. To explore this idea further, future studies may wish to combine clinical and pre‐clinical models to fully explore a possible neural link between ASD and AN.

Finally, recent work in the field of autism has suggested that variation in grey matter and grey matter reduction in the cerebellum are related to ASD traits (D'Mello & Stoodley, 2015; Mei et al., 2020). Work from the EU‐AIMS longitudinal European Autism project using 347 autistic individuals found decreased densities in a number of brain regions and that grey matter variation in a number of widespread regions was related to ASD symptoms (Mei et al., 2020). This led the authors to conclude that widespread variation, rather than localised changes, underpin autistic symptoms (Mei et al., 2020). Grey matter reductions have also commonly been reported in cerebellar areas such as crus I, lobule VIII and IX in autistic individuals and these areas are linked to specific ASD phenotypes (D'Mello & Stoodley, 2015). This study did not examine variation amongst structural measures and links to ASD traits, or examine group‐based structural differences in the cerebellum due to limitations on Freesurfer's surface‐based stream. Further supporting this idea, previous works in AN, using structural covariance networks, have found a less densely distributed connective organization (Collantoni et al., 2017, 2019) possibly displaying a similar pattern in autism (Mei et al., 2020). It may be the case that ASD traits in AN are linked to global variation rather than directly to the global measures. Functional imaging in AN has also implicated networks involving the left cerebellum (Gaudio et al., 2018). Therefore, it could be that group‐based cerebellum differences exist which are associated to ASD characteristics, which this study could not examine. Future studies may then wish to examine covariance of measures as well as the cerebellum and any possible links to ASD traits.

Comparing our study to previous work examining ASD traits and structure in AN is extremely difficult as this is an under‐researched area. To the best of our knowledge, only one study has reported structural differences, finding a negative correlation between the left superior temporal sulcus and ASD characteristics in individuals with AN (Björnsdotter et al., 2018). We were unable to replicate previous results. This could be due to numerous reasons. First, our study and Björnsdotter and colleagues used different analysis methods and different hypotheses were formulated. This study, building on previous research showing differences in surface‐based measures in AN (Bernardoni et al., 2016, 2018; Leppanen et al., 2019) analysed surface based topology, while Björnsdotter and colleagues building on work looking at localised volume changes (Brooks et al., 2011; Fonville, et al., 2013) examined localised volume (Björnsdotter et al., 2018). Though volume is related to surface‐based measures it is however an independent measure and different to other measures (Winkler et al., 2010) and this may explain why we were unable to replicate Björnsdotter and colleagues' findings. Another possible reason could be that previous work may have been underpowered with a smaller sample size which raises the possibility of false positive findings (Vadillo et al., 2016). Finally, different measures of ASD traits were used in the different studies, with the ADOS‐2 being used for the current study and previous work using the AQ (Bernardoni et al., 2016). The ADOS‐2 is considered a ‘gold standard’ assessment (Kerr‐Gaffney et al., 2021) while some authors have criticised the AQ suggesting that co‐morbidities such as depression can cause false positives and those without co‐morbidities being misclassified, causing false negatives (Ashwood et al., 2016). This is perhaps another reason why we were unable to replicate previous work.

Our study also produced a number of interesting findings and null results unrelated to ASD correlations which deserve consideration. Though we replicated localised LGI changes, we were unable to replicate widespread reductions in CC, CT and LGI seen in previous studies (Bernardoni et al., 2016, 2018, 2016; King et al., 2015). A possible reason for this may be that the BMI of our participants was on average higher than in previous studies. Previous work has examined underweight AN participants with an average BMI of 14.5 (King et al., 2015) and 14.8 (Bernardoni et al., 2016, 2018) compared to the average BMI of our AAN group of 16.38. According to the Royal College of Paediatrics and Child health, the age‐adjusted BMI of participants in previous work is between −4 standard deviations and the first percentile, while the age‐adjusted BMI for our AAN group would be between first and second percentile (Royal College of Paediatrics and Child Health, 2013). This demonstrates that even after taking age into account, our participants on average had higher BMI than in previous work. Previous work has highlighted the importance of BMI and cortical measures with Lavagnino and colleagues demonstrating a positive correlation between BMI and CT in a number of brain regions in underweight individuals with AN (Lavagnino et al., 2016). In addition to this, longitudinal studies have highlighted the importance of change in BMI as driving CT, CC and LGI differences, demonstrating that BMI rising from 14.8 to 18.7/18.8 normalises changes (Bernardoni et al., 2016, 2018). If, as previous work suggests, BMI is a main driving force in structural changes then it could perhaps be that our underweight group's BMI was not significantly low enough to cause the widespread changes seen in previous work. There is some evidence for this theory as Lavagnino and colleagues also found no group‐based differences in CT in participants of a similar age and BMI (Lavagnino et al., 2016). A potential mechanism for this could come from structural covariance studies, which suggest that AN impacts global covariation patterns (Collantoni et al., 2017, 2019). Therefore it could be that the localised changes found in this and in previous work represent a ‘tip of the iceberg’ phenomena, the beginning of the results of starvation on global covariation patterns. This tentative hypothesis would need to be explored further with longitudinal and structural covariance studies, in particular exploring the link between structural covariance and BMI, which to the best of our knowledge has yet to be done.

The other interesting result was the difference in global CT between the AAN and WR groups. This is an unusual result and most likely reflects a difference in age rather than illness state. Though a group difference was found with age, post‐hoc tests couldn't determine the direction of significance, however the mean age of AAN is slightly higher compared to WR individuals, even if that did not reach statistical significance. We are therefore cautious to over interpret this finding and suggest that if this finding is replicated in future work then it would of course need to be examined further.

This brings us onto the limitations of the study. Our first major limitation was that we could not fully control for the effect of age in our models. The addition of age to the GLM caused significant collinearity with the group variables (presented in the supplementary material) which has been shown to reduce the power of the analysis and make parameter estimates unreliable (Mumford et al., 2015). This is most likely due to the addition of age in group stratification, meaning that age and group exhibit collinearity. Although the use of age in group selection has potentially confounding effects in the model it is critical to incorporate when using participants under the age of 18. This is because the BMI formula does not take age into account, so if BMI alone is a major criteria for group selection, then oddities such as a 13 year old with a BMI of 18 being labelled as underweight (despite this being in the healthy weight range for their age) occur. It should also be noted that due to the aim of this study, to examine ASD traits and their correlation to structure in AN, age related effects are less of a confounding factor than if this study was a pure replication study or attempting to find new group based structural differences. Despite this, future studies should try and control for age as much as possible when examining structure.

Another limitation is the heterogeneity of the participants and a lack of an ASD group to directly compare to. The WR group included a heterogeneous mix of fully recovered and symptomatic participants and duration of weight‐restoration was unknown, these may affect our findings. Also the AAN group was recruited from a variety of services and it was unclear if some in this group were engaged in treatment and improving or deteriorating in nutritional status. Underweight individuals engaging with treatment will be at a different stage of the disorder compared to underweight individuals who are not. AN participants were also at varying stages of treatment leading to a heterogeneous mix of treatments. Theses may have all introduced heterogeneity into the groups which may possibly explain some of our findings. We also did not have knowledge of hydration and nutritional status on the day of the MRI scan, which may potentially confound our results. Finally, not having a group of ASD individuals to directly compare with limits this studies ability to elicit possible trans‐diagnostic structural links between AN and ASD. We would therefore recommend that future studies control for heterogeneity of the groups, including hydration and nutritional status on the day of the MRI scan and contain a group of ASD individuals to allow for a direct comparison.

Our final limitation of the study is that we could not control for medication and AN diagnostic subtype in the analysis. Different subtypes of AN have been shown to have structural differences, possibly reflecting different aspects of food intake (Brooks et al., 2011). Also the use of medication, in particular anti‐psychotics, has been demonstrated to effect structural measures in depression (Voineskos et al., 2020). It should be noted however, that studies which have compared medicated and non‐medicated participants in AN have shown no difference (Fonville et al., 2013; Leppanen et al., 2019). Still we would recommend that future research control for the effects of medication and AN subtype on structural measures.

Despite these limitations, this study contributes to an under‐researched area, investigating the neural link between AN and ASD traits. Though no link between structure and ASD traits was found, this study offers possible avenues for future work such as the exploration of global covariance and ASD traits as well as the importance of longitudinal studies. As well as adding to the extremely limited previous work in this field, this study highlights the importance of future work in this area.

## CONFLICT OF INTEREST

None.

## ETHICAL STANDARDS

The authors assert that all procedures contributing to this work comply with the ethical standards of the relevant national and institutional committees on human experimentation and with the Helsinki Declaration of 1975, as revised in 2008.

## Supporting information

Supporting Information S1

Figure S1

## Data Availability

The data that support the findings of this study are available from the corresponding author upon reasonable request.
