# Supplementary material for: Examining the relationship between autistic spectrum disorder characteristics and structural brain differences seen in anorexia nervosa
Source: Eur Eat Disord Rev. 2022 May 15;30(5):459–73. doi: 10.1002/erv.2910 (PMC9546313; doi:10.1002/erv.2910)
Supplement: Supplementary file 1 — Supporting Information S1 [file ERV-30-459-s002.docx]

**Table 1. Correlation and P values Between Cortical Volumes and Clinical Measures.**

| Volume | Variable | AAN  p(FWE) Rho | | WR  p(FWE) Rho | | HC  p(FWE) Rho | |
| --- | --- | --- | --- | --- | --- | --- | --- |
| Gray Matter Volume | Illness Duration | 1.00 | -0.16 | 0.83 | -0.34 | - | - |
|  | EDE-Q | 1.00 | 0.07 | 1.00 | 0.04 | 1.00 | 0.05 |
|  | ADOS-2 Communication and Social | 1.00 | 0.02 | 0.92 | 0.35 | 1.00 | -0.28 |
|  | ADOS-2 Creativity | 1.00 | -0.01 | 1.00 | 0.27 | 1.00 | -0.01 |
|  | ADOS-2 Stereotyped and repetitive | 1.00 | 0.30 | 1.00 | 0.23 | 1.00 | -0.18 |
|  | BMI | 1.00 | 0.24 | 1.00 | -0.12 | 0.98 | -0.27 |
|  | Age | 1.00 | -0.19 | 0.56 | -0.35 | 1.00 | -0.04 |
| White Matter  Volume | Illness Duration | 1.00 | -0.11 | 1.00 | -0.24 | - | - |
|  | EDE-Q | 1.00 | 0.23 | 1.00 | 0.12 | 1.00 | 0.23 |
|  | ADOS-2 Communication and Social | 1.00 | -0.06 | 1.00 | 0.28 | 0.96 | -0.36 |
|  | ADOS-2 Creativity | 1.00 | 0.27 | 1.00 | 0.25 | 1.00 | -0.10 |
|  | ADOS-2 Stereotyped and repetitive | 1.00 | 0.24 | 1.00 | 0.15 | 1.00 | 0.03 |
|  | BMI | 1.00 | 0.20 | 1.00 | 0.08 | 1.00 | -0.05 |
|  | Age | 1.00 | 0.17 | 1.00 | -0.16 | 0.53 | 0.33 |
| Thickness | Illness Duration | 1.00 | -0.13 | 1.00 | -0.21 | - | - |
|  | EDE-Q | 1.00 | -0.11 | 1.00 | -0.10 | 1.00 | -0.12 |
|  | ADOS-2 Communication and Social | 1.00 | 0.06 | 1.00 | -0.11 | 1.00 | 0.31 |
|  | ADOS-2 Creativity | 1.00 | -0.09 | 1.00 | -0.11 | 1.00 | 0.05 |
|  | ADOS-2 Stereotyped and repetitive | 1.00 | 0.24 | 1.00 | 0.13 | 1.00 | -0.10 |
|  | BMI | 0.99 | 0.28 | 1.00 | -0.06 | 0.99 | -0.26 |
|  | Age | 0.96 | -0.29 | 0.99 | -0.26 | 0.23 | -0.36 |
| Curvature | Illness Duration | 1.00 | 0.18 | 1.00 | 0.02 | - | - |
|  | EDE-Q | 1.00 | 0.01 | 1.00 | 0.02 | 1.00 | -0.18 |
|  | ADOS-2 Communication and Social | 1.00 | 0.03 | 1.00 | -0.07 | 0.98 | 0.34 |
|  | ADOS-2 Creativity | 0.98 | -0.35 | 1.00 | -0.12 | 1.00 | -0.22 |
|  | ADOS-2 Stereotyped and repetitive | 1.00 | -0.20 | 1.00 | 0.01 | 1.00 | -0.20 |
|  | BMI | 1.00 | -0.17 | 1.00 | -0.20 | 1.00 | -0.05 |
|  | Age | 1.00 | -0.04 | 1.00 | 0.03 | 0.24 | -0.36 |
| LGI | Illness Duration | 1.00 | -0.02 | 1.00 | -0.13 | - | - |
|  | EDE-Q | 1.00 | 0.04 | 1.00 | 0.11 | 1.00 | -0.01 |
|  | ADOS-2 Communication and Social | 1.00 | -0.03 | 1.00 | 0.21 | 1.00 | -0.20 |
|  | ADOS-2 Creativity | 0.99 | -0.34 | 1.00 | 0.12 | 1.00 | -0.07 |
|  | ADOS-2 Stereotyped and repetitive | 1.00 | 0.03 | 1.00 | 0.10 | 1.00 | -0.06 |
|  | BMI | 1.00 | 0.06 | 1.00 | -0.21 | 0.90 | -0.30 |
|  | Age | **< 0.01** | -0.54 | 1.00 | -0.19 | 1.00 | -0.8 |
| Area | Illness Duration | 1.00 | -0.18 | 1.00 | -0.24 | - | - |
|  | EDE-Q | 1.00 | 0.19 | 1.00 | 0.05 | 1.00 | 0.12 |
|  | ADOS-2 Communication and Social | 1.00 | -0.06 | 0.53 | 0.41 | 0.95 | -0.37 |
|  | ADOS-2 Creativity | 1.00 | 0.05 | 0.96 | 0.34 | 1.00 | -0.10 |
|  | ADOS-2 Stereotyped and repetitive | 1.00 | 0.06 | 1.00 | 0.22 | 1.00 | -0.12 |
|  | BMI | 1.00 | 0.08 | 1.00 | -0.08 | 1.00 | -0.19 |
|  | Age | 1.00 | -0.08 | 1.00 | -0.21 | 1.00 | 0.10 |
| Abbreviation: ADOS-2 = Autism Diagnostic Observation schedule, 2^nd^ edition ; AAN = Actuely Underweight Anorexia Nervosa group; BMI= Body Mass Index; HC = Healthy Controls; LGI= Local Gyrification Index; WR= Weight-Restored Anorexia Nervosa Group. | | | | | | | |

**Table 3. Correlation and P values between Right LGI post-central gyrus cluster located at 38.8, -16.7,-8.5 and clinical measures.**

| Variable | AAN  p(FWE) Rho | | WR  p(FWE) Rho | | HC  p(FWE) Rho | |
| --- | --- | --- | --- | --- | --- | --- |
| Illness Duration | 1.00 | -0.04 | 1.00 | -0.04 | - | - |
| EDE-Q | 1.00 | 0.12 | 0.99 | -0.20 | 0.99 | -0.18 |
| ADOS-2 Communication and Social | 0.99 | 0.14 | 0.76 | 0.31 | 1.00 | 0.12 |
| ADOS-2 Creativity | 0.99 | -0.24 | 1.00 | 0.11 | 1.00 | -0.13 |
| ADOS-2 Stereotyped and repetitive | 1.00 | 0.09 | 0.96 | 0.26 | 1.00 | -0.18 |
| BMI | 1.00 | -0.03 | 0.59 | -0.29 | 0.46 | -0.30 |
| Age | **0.02** | -0.45 | 0.12 | -0.38 | 0.31 | -0.31 |
| Abbreviation: ADOS-2 = Autism Diagnostic Observation schedule, 2^nd^ edition ; AAN = Actuely Underweight Anorexia Nervosa group; BMI= Body Mass Index; HC = Healthy Controls; WR= Weight-Restored Anorexia Nervosa Group. | | | | | | |

**Table 4. Correlation and P values between right LGI supramarginal gyrus cluster and clinical measures.**

| Variable | AAN  p(FWE) Rho | | WR  p(FWE) Rho | | HC  p(FWE) Rho | |
| --- | --- | --- | --- | --- | --- | --- |
| Illness Duration | 1.00 | -0.04 | 1.00 | 0.03 | - | - |
| EDE-Q | 1.00 | 0.12 | 1.00 | -0.09 | 1.00 | -0.03 |
| ADOS-2 Communication and Social | 1.00 | 0.04 | 0.36 | 0.38 | 1.00 | -0.12 |
| ADOS-2 Creativity | 1.00 | -0.08 | 1.00 | 0.09 | 1.00 | 0.07 |
| ADOS-2 Stereotyped and repetitive | 1.00 | 0.07 | 0.99 | 0.24 | 1.00 | 0.08 |
| BMI | 1.00 | 0.08 | 1.00 | -0.03 | 1.00 | -0.12 |
| Age | **0.01** | -0.46 | 0.71 | -0.27 | 1.00 | -0.08 |
| Abbreviation: ADOS-2 = Autism Diagnostic Observation schedule, 2^nd^ edition ; AAN = Actuely Underweight Anorexia Nervosa group; BMI= Body Mass Index; HC = Healthy Controls; WR= Weight-Restored Anorexia Nervosa Group. | | | | | | |

**Table 5. Variance Influence Factor from vertex models.**

| Model with age | | Model without Age | |
| --- | --- | --- | --- |
| Feature | VIF | Feature | VIF |
| Age | 2.37 | - | - |
| AAN | 12.14 | AAN | 1.00 |
| WR | 11.21 | WR | 1.00 |
| HC | 14.31 | HC | 1.00 |
| Voxels | 1.00 | Voxels | 1.00 |
| Abbreviation: AAN = Actuely Underweight Anorexia Nervosa group; HC = Healthy Controls; WR= Weight-Restored Anorexia Nervosa Group; VIF = Variance Influence Factor | | | |

fig 4 here
