# Supplementary figures and images for: Examining the relationship between autistic spectrum disorder characteristics and structural brain differences seen in anorexia nervosa
Source: Eur Eat Disord Rev. 2022 May 15;30(5):459–73. doi: 10.1002/erv.2910 (PMC9546313; doi:10.1002/erv.2910)

Heatmap with age in model

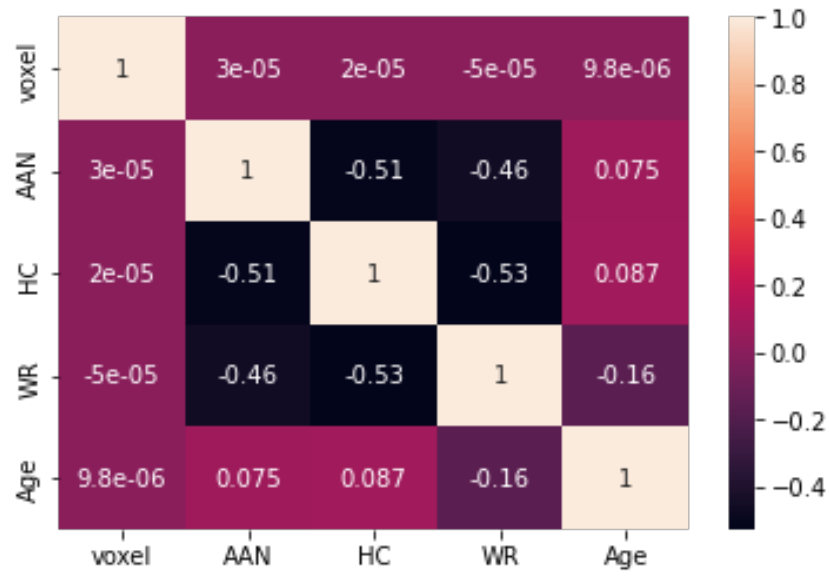

Heatmap without age in model

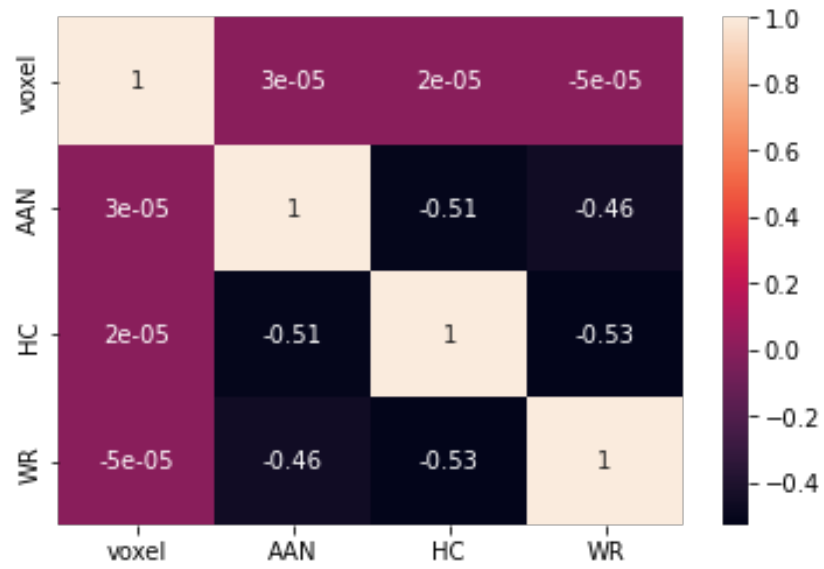

Supplement: Supplementary file 2 — Figure S1 [file ERV-30-459-s001.pdf]
